# Extensible byssus of *Pinctada fucata*: Ca^2+^-stabilized nanocavities and a thrombospondin-1 protein

**DOI:** 10.1038/srep15018

**Published:** 2015-10-08

**Authors:** Chuang Liu, Shiguo Li, Jingliang Huang, Yangjia Liu, Ganchu Jia, Liping Xie, Rongqing Zhang

**Affiliations:** 1 Institute of Marine Biotechnology, Collaborative Innovation Center of Deep Sea Biology, School of Life Sciences, Tsinghua University, Beijing 100084 China; 2 Tsinghua-Peking Joint Center for Life Sciences, School of Life Sciences, Tsinghua University, Beijing 100084 China

## Abstract

The extensible byssus is produced by the foot of bivalve animals, including the pearl oyster *Pinctada fucata,* and enables them to attach to hard underwater surfaces. However, the mechanism of their extensibility is not well understood. To understand this mechanism, we analyzed the ultrastructure, composition and mechanical properties of the *P. fucata* byssus using electron microscopy, elemental analysis, proteomics and mechanical testing. In contrast to the microstructures of *Mytilus sp.* byssus, the *P. fucata* byssus has an exterior cuticle without granules and an inner core with nanocavities. The removal of Ca^2+^ by ethylenediaminetetraacetic acid (EDTA) treatment expands the nanocavities and reduces the extensibility of the byssus, which is accompanied by a decrease in the β-sheet conformation of byssal proteins. Through proteomic methods, several proteins with antioxidant and anti-corrosive properties were identified as the main components of the distal byssus regions. Specifically, a protein containing thrombospondin-1 (TSP-1), which is highly expressed in the foot, is hypothesized to be responsible for byssus extensibility. Together, our findings demonstrate the importance of inorganic ions and multiple proteins for bivalve byssus extension, which could guide the future design of biomaterials for use in seawater.

In recent years, marine animals have attracted considerable attention in various areas of bioinspired engineering^1^,^2^,^3^,^4^. Marine animals must adapt rapidly to dynamic environmental variables, including temperature and salinity. To adapt to these changing factors, mollusca bivalvia have developed various strategies, such as remarkably tough calcified shells^5^ and byssus with underwater extensibility and adhesivity^6^.

Byssus is a fibrous polymer produced by marine mollusca bivalvia to secure themselves to a solid surface through adhesion and extension. Previous studies have shown that the dopa-rich mussel foot proteins 2 (mfp2), mfp3, mfp5, and mfp6 and the metal ions in *Mytilus* species contribute to adhesion^6^,^7^. Consequently, mussel-inspired catechol chemistry is emerging as a powerful approach for developing coatings for various substrates^3^,^8^. Byssal precollagens (PreCOLs) represent a family of proteins that self-assemble to form the extensible part of the byssus. In the proximal portion, the main collagenous protein is preCol-P, while the distal one mainly contains preCol-D, which are gradually distributed along the thread. By contrast, preCol-NG uniformly distributed in the entire thread^9^. Mfp1 is the main macromolecular component of the cuticle, and its chelation with Fe^3+^ has been linked to the extensibility of the byssus^6^. Studies of these proteins have inspired the development of self-healing materials^10^,^11^. In addition to identifying its component biomolecules^12^, elucidating the nanoscopic structures of its biological constituents is vital for understanding the function of biomaterials^13^. Studies have revealed that in *Mytilus*, denser cross-linking through metal coordination with 3,4-dihydroxyphenylalanine (dopa) in the granules accounts for byssus hardness, whereas less cross-linked protein in the granules contributes to byssus extensibility^3^,^4^. However, rare reports have shown that byssus from different bivalvia species may use a variety of mechanisms to create extensibility. For example, the elastic proteins in the byssus of the giant clam (*Tridacna maxima*) form a stable rope-like structure containing tetrameric coiled-coil α-helices^14^.

Here, we showed the importance of inorganic ions and byssal proteins for byssus extensibility in the bivalvia pearl oyster *Pinctada fucata*. Through ultrathin sectioning and electron microscopy, we showed that the cuticle of byssu*s* is composed of compact protein fibers, whereas the core is composed of loose protein nanofibers with nanocavities. Moreover, Fourier transform infrared spectroscopy (FTIR) and mechanical tests were performed to investigate the effect of Ca^2+^ on byssal protein conformation and the mechanical properties of the byssus, respectively. Finally, proteomic data identified major biomolecules in the byssal proteins. Among them, a thrombospondin-1 (TSP-1) is proposed to be accountable for the extensibility of the byssus based on sequence analysis and real-time PCR.

## Methods

All methods were carried out in accordance with the approved guidelines. All experimental protocols were approved by the Animal Experimental Ethics Committee of Tsinghua University, Beijing, China.

### Sample preparation

The pearl oyster *P. fucata* (with shells 5.5–6.5 cm in length and 30–40 g of wet weight, at approximately 2 years of age) was obtained from the Zhanjiang Pearl Farm (Guangxi Province, China). In our laboratory, oysters were kept at approximately 20 °C in an aquarium that contained aerated artificial seawater at 3% salinity. The byssus was removed from oysters without injuring the organism and was washed thoroughly in distilled water. The native byssus was stored at 4 °C in artificial water prior to experimental testing.

### Characterization

#### Scanning electron microscopy (SEM)

To observe the microscale ultrastructure of the byssus, SEM was performed. The fresh byssus was embedded in PEG-2000 (Sigma), and 10 μm sections were cut on a Leica CM1900 microtome at −20 °C from the distal to proximal region. The sections were washed thoroughly to remove any remaining PEG and were subsequently transferred to a quartz slide. For SEM (FEI Quanta 200, 15 keV), the samples were first vacuum freeze-dried at −40 °C (Christ alpha 1–2) and were then sputter-coated with a nanoroughened film of gold for 60 seconds. For light microscopy, the aforementioned cut samples were viewed on an Olympus BX 60 equipped with a camera.

#### Transmission electron microscopy (TEM)

To observe the nanoscale ultrastructure of the byssus, TEM was conducted. The fresh byssus was fixed in 2.5% glutaraldehyde for 2 h followed by 1% osmic acid for 2 h. They were dehydrated in serial ethanol solutions (50, 70, 80, 90, 100%), followed by 1:1 ethanol:acetone and finally 100% acetone. Samples were then embedded in Spurrs resin and polymerized at 60 °C for 48 h. Subsequently, they were sectioned on a microtome (Leica EM UC6) to produce 80 nm thin transverse sections. Finally, the samples were stained with 3% mixed uranyl acetate and lead citrate for 10 min. Micrographs were obtained using a Hitachi Limited H-7650 transmission electron microscope operated at 80 kV. Ethylenediaminetetraacetic acid (EDTA) treatment was conducted in both the whole thread and thin sections. For the whole thread, they were incubated in 10, 200 and 500 mM EDTA, 10 mM Tris at pH 5.0 for 12 h. Ca^2+^ restoring experiments were performed by immersing the above 200 mM EDTA-treated threads in 20 mM CaCl_2_ and stirring for 12 h. For thin sections of byssus, only 200 mM EDTA was used, and the sections were immersed on the grid for 10 min.

#### Inductively coupled plasma (ICP)

To determine the overall concentration of the elements of the distal byssus regions by ICP, at least five pristine or 200 mM EDTA-treated samples were thoroughly rinsed with DI water and then vacuum freeze-dried at −40 °C before being dissolved in a nitric acid solution.

#### X-ray photoelectron spectroscopy (XPS)

The surface elements of at least five samples were measured on a Perkin-Elmer PHI-5300 Quantum X-ray photoelectron spectrometer with monochromatic Al K-alpha radiation (1486.7 eV), and the binding energies were normalized to the C1s peak at 284.8 eV.

#### Fourier transform infrared spectroscopy (FTIR)

The conformation of air-dried byssus was acquired by attenuated total reflectance Fourier transform infrared spectroscopy (ATR-FTIR) using a Bruker Vertex 70 spectrometer. All spectra were recorded with a resolution of 2 cm^−1^, and peaks at approximately 1700–1580 cm^–1^ were decomposed with PeakFit 4.12 software, with R^2^ greater than 0.99. Baselines were corrected by linear background subtraction with 3% tolerance. The number of Gaussians were decided according to previous reports on similar protein materials^10^,^15^,^16^. Local minima in the second derivative were used to identify peaks in the amide I region, which were fit as Gaussian curves. The peak position, height, and width were allowed to vary in a small range during an iterative least-squares method to minimize the residuals between reconstituted spectra and the experimental spectra^15^. In this case, the amine I region was deconvoluted to five curves, in which unknown region, β-sheet, unordered region, α-helix and β-turn structures were centered at wavenumbers of approximately 1612, 1628, 1643, 1660 and 1679 cm^-1^, respectively.

### Mechanical performance of the distal regions of threads

To characterize the mechanical properties of the byssus, the distal byssus in three states (native, 200 mM EDTA treated and Ca^2+^ restored byssus) were tested on a tensile testing instrument (Zwick Roell Z005). The thread was clamped firmly across the testing sections to prevent slippage. The initial length of the samples was 12 mm, and the strain rate was 0.5 mm/min at resolution of 10 μm. The test was performed at room temperature, and the samples were kept in deionized (DI) water until the test. For each condition, five samples were tested.

### Extraction of foot proteins and proteomic analysis

To identify the component proteins of the byssus, a proteomic analysis was conducted. Byssus was collected from oyster and was rinsed with DI water to remove any salt. The distal regions were powdered in liquid nitrogen and were homogenized using a DHS tissue grinder on ice with 5% acetic acid and 8 M urea^17^. Insoluble proteins were harvested by centrifugation (20 000 g, 4 °C, 30 min) and then boiled in a solution of 10 mM dithiothreitol (DTT), 20 mM SDS, and 40 mM Tris-HCl for 30 mins. Finally, the boiled solution was subjected to 12% SDS-PAGE and stained with Coomassie Brilliant Blue 250. Protein bands were selected and digested by trypsin. The masses and charges of the digested peptides were analyzed on a Thermo Scientific LTQ Orbitrap Velos mass spectrometer with a Dionex U-3000 Rapid Separation nano LC system. The LC-MS/MS data were searched against the *P. fucata* predicted protein database (http://marinegenomics.oist.jp/genomes)^18^ by a Mascot 2.1 search engine with carbamidoethylated cysteine as a fixed modification and oxidized methionine and tryptophan as variable modifications. Proteins with scores ≥5.0 were identified using Blastp and tBlastn searches against the NCBI database (http://blast.ncbi.nlm.nih.gov/Blast.cgi). Functional domains were analyzed by SMART (http://smart.embl-heidelberg.de/). Repeat sequences were analyzed using RADAR (http://www.ebi.ac.uk/Tools/pfa/radar/). Three-dimensional structures of the proteins were predicted using Phyre 2^19^. The generated sequences were deposited at GenBank KP737361-KP737375.

### Tissue-specific gene expression analysis by real-time PCR

Real-time PCR was conducted to quantify gene expression levels. Total RNA from the gonad, muscle, foot, mantle and gill was extracted using TRIzol^®^ Reagent (Life Technologies^TM^), and first-strand cDNA was synthesized using the GoScript^TM^ Reverse Transcription System (Promega)^20^,^21^. A typical reaction contains the following: SYBR^®^ Premix Ex Taq^TM^ II(Takara) 12.5 μL, forward primer 0.5 μL, reverse primer 0.5 μL, cDNA template 0.5 μL and H_2_O 6 μL. The PCR parameters were as follows: 95 °C for 30 s (1 cycle); 95 °C for 5 s, 60 °C for 30 s (40 cycles), 72 °C for 35 s. Dissociation curves were investigated to determine product purity and amplification specificity. Relative gene expression levels were calculated using the delta-delta method: fold = 2^−[ΔCt sample−ΔCt calibrator]^ = 2^−ΔΔCt^. Here, the “ΔCt calibrator” represents the mean ΔCt value of β-actin (GeneBank accession: AF378128.1) in the corresponding tissue because β-actin has relatively stable expression levels^20^.

The primers used were as follows: TSP-1(5′-AAGAAACGGAGGCACAAACTG-3′;

5′-CTGTCGTCTGGAGCACTGAAT-3′).

Actin(5′-CACAGCATTCATACAAGCAAAGG-3′;

5′-TGGGGCATCGTCTCCTCCAAAC-3′).

### Image statistics process

Images were processed using ImageJ, first by transferring images to binary and adjusting the threshold to fit the cavities, then by using the “analyzing particles” function to calculate areas. For each condition, three images with approximately 200 cavities each were analyzed for size distribution. Statistical comparisons were performed with one-way ANOVA by using Origin 8.0 software.

## Results and Discussion

### The structure of the byssus

**Figure 1:**
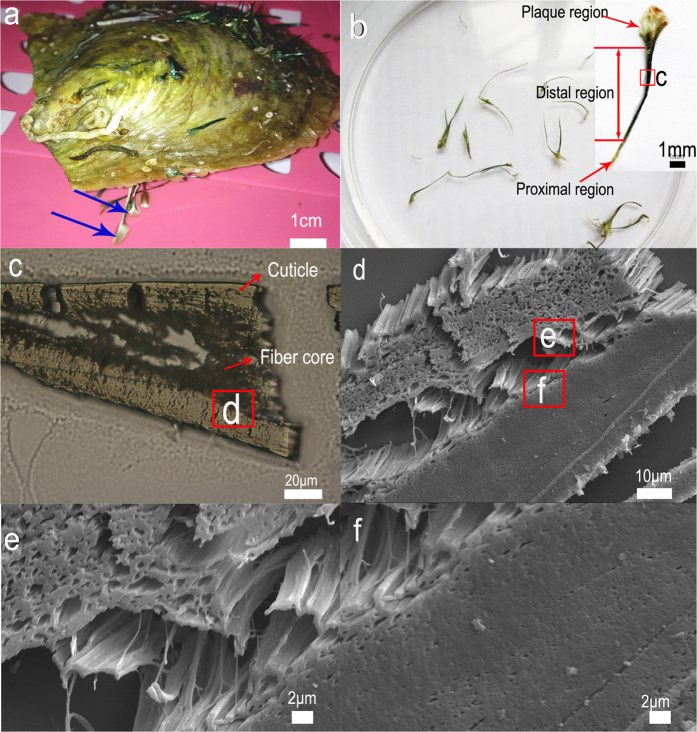
(**a**) The byssus (blue arrows) of *P. fucata* attaches to the glass of a tank. (**b**) The three portions of the byssus: the proximal, distal and plaque regions. (**c**) Optical photograph of a transverse cross-section of the distal region. (**d**) SEM of a transverse cross-section of the distal region. (**e**) High magnification shows the protein fibers in the core. (**f**) High magnification shows the compact cuticle region.

*P. fucata* anchor themselves onto almost any surface, including glass, rock, plastic or other shells, through the byssus (Fig. 1a), which typically consists of 3–10 threads 1–2 cm in length (Fig. 1b). In comparison, *Mytilus edulis* typically has 50–100 threads, each 4–5 cm in length^22^. The macroscopic appearance of the *P. fucata* byssus features similarities with mussel byssus^23^,^24^ and can be classified into three distinct regions: proximal, distal and plaque (Fig. 1b). However, we found that its microscopic structures differed substantially. The proximal region was composed of foam-like granules with hollow spaces (Figure S1), which did not exist in the mussel^6^. The distal region of the byssus occupies approximately 80% of the total thread length and is responsible for its extensibility. Therefore, to obtain insights into byssus extensibility, we concentrated on the distal region for the remainder of this study (Fig. 1b).

SEM of transverse cross-sections of the distal region and the increased magnification image revealed a two-layer structure (Fig. 1c,d). The core was composed of nanoscale protein fibers aligned parallel to the axis of byssus, with submicron-scale areas where electrons are easy to transmit, termed “nanocavities” between the fibers (Fig. 1e). The 20–50 μm cuticle, by comparison, was compact (Fig. 1f). Unlike the mussel’s cuticle, granules with diameters of approximately 200 nm in *Mytilus californianus*^25^ or 800 nm in *Mytilus galloprovincialis* were not found^26^. The granules have been proven to be linked to extensibility and cuticle hardness^2^. The difference between the cuticle structures of the two species implied a different mechanism for byssus extensibility.

### Nanocavities in the byssus

**Figure 2:**
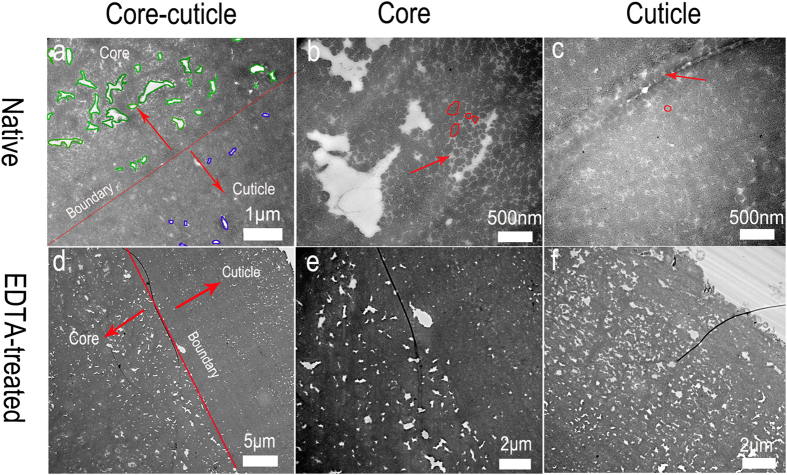
TEM images of transverse cross-sections of distal regions. (**a**) High magnification shows the boundary between the core and cuticle of native byssus (green and blue areas are nanocavities in the core and cuticle, respectively). (**b**) The core has cavities between protein fibrils (red circles). (**c**) The cuticle is composed of compact protein fibrils (red circles; red arrows in b and c are matrix proteins between proteins fibrils) (**d**) TEM of transverse cross-sections of distal regions treated with 200 mM EDTA in thin sections; (**e**) and (**f**) show the core and cuticle with expanded cavities.

As mentioned above, knowledge of ultrastructure is critical for understanding the action of a biomaterial. Therefore, TEM images of ultrathin (80 nm) transverse sections of byssus were taken and revealed nanocavities with different void sizes in both the core and cuticle (Fig. 2a). Holes with sharp edges and bright contrast were attributed to incomplete infiltration of the resin (Figure S2). Therefore, the observed nanocavities are intrinsic to the byssus of *P. fucata* rather than a drying artifact caused by the sample preparation process. Previous studies of *Nephila* spider silk found similar nanocavities by using a cryomicrotome^27^,^28^. The nanocavities separated clusters of 40–180 nm protein fibers (Fig. 2b,c), which were connected by matrix proteins, as indicated by lighter electron contrast (red arrows in Fig. 2b,c). In *M. galloprovincialis*, the byssus contained thread matrix proteins (TMPs) and the proximal thread matrix protein 1 (PTMP1)^29^, which acted as a coupling agent between collagens and other byssal components^9^. However, the thread matrix proteins in *P. fucata* have not yet been identified.

Metals were previously proposed to participate in linking byssal proteins through histidine-metal, cysteine-metal or dopa-metal interactions^30^. To examine the roles of metals in the byssus, they were removed by chelation with EDTA in both the whole thread and thin sections. Surprisingly, after immersing the thin section of byssus on the grid in EDTA for 10 minutes, the cavities in the core (Fig. 2d,e) and cuticle (Fig. 2d,f) expanded, and the initial cavity sizes of over 0.025 μm^2^ increased to 70% and 50% in the core and cuticle regions, respectively (Table 1). The expansion was more obvious if the whole thread had been immersed in EDTA (Figure S3 and S4). In the native byssus, by contrast, the cavities in the core and cuticle with initial sizes of over 0.025 μm^2^ were 50% and 0%, respectively (Table 1).

**Figure 3:**
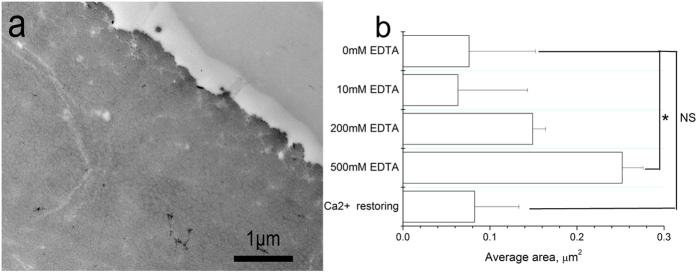
(**a**) TEM image of transverse cross-sections of the distal region treated with 200 mM EDTA for 12 h followed by immersion in CaCl_2_ for 12 h. (**b**) A comparison of the average areas in the byssus core under different conditions (three samples with 200 cavities each were analyzed for the size distribution. One-way ANOVA tests are performed, for 0 mM EDTA sample and 500 mM EDTA treated sample, P < 0.05; for 0 mM EDTA sample and Ca^2+^ restoring sample, P > 0.05, NS = not significant).

To confirm that the expanded cavities were caused by loss of metals, a Ca^2+^ restoring experiment was conducted. The enlarged cavities in the EDTA-treated byssus returned to their original size after the Ca^2+^ restoring treatment (Fig. 3a). Furthermore, with an increase in EDTA concentration from 10, 200 to 500 mM, the average size of the cavities in the core of byssus increased (Fig. 3b). These results demonstrate that loss of metals disturbs the fibril structure, resulting in reversibly expanded cavities. The expanded cavities may partially be ascribed to the loss of calcium volume. However, estimation of the volume of cavities in the byssus is hard due to the number of cavities is not accountable.

### Conformational change of byssal proteins by FTIR

**Figure 4:**
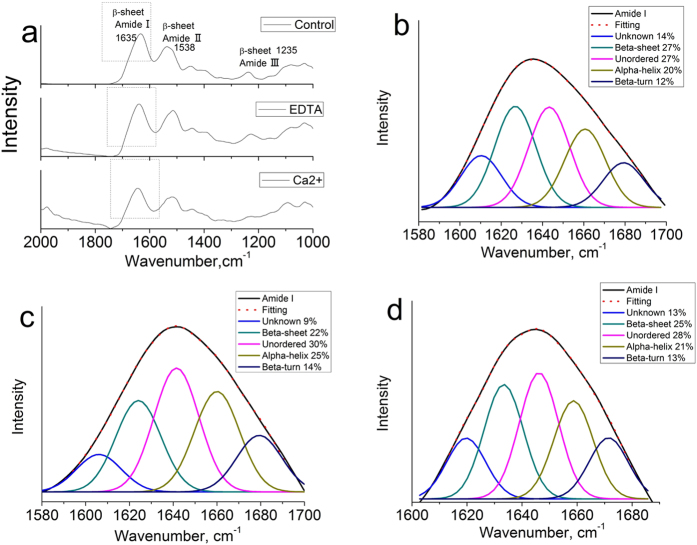
(**a**) FTIR spectra of native, 200 mM EDTA-treated, and 20 mM Ca^2+^ restored byssus of *P. fucata.* Amide I region decomposition of native byssus (**b**), 200 mM EDTA-treated byssus (**c**), and 20 mM Ca^2+^ restored byssus (EDTA was removed and replaced with an excess volume of 20 mM Ca^2+^) (**d**).

To investigate conformational changes caused by EDTA treatment, three groups of dried byssus were tested: native byssus as the control, EDTA-treated byssus and Ca^2+^ restored byssus after EDTA treatment. All three samples had absorbances ranging from 1700 to 1585 cm^−1^, 1585–1490 cm^−1^ and 1270–1210 cm^−1^, corresponding to the amide I, amide II and amide III regions of proteins (Fig. 4a). Protein secondary structures were obtained by peak deconvolution in the amide I regions^10^,^15^. The native byssus was composed of 27% β-sheet, 27% unordered region, 20% α-helix, 12% β-turn and 14% unknown structure according to their contribution to amide I absorbance (Fig. 4b). After EDTA treatment, the byssus exhibited 22% β-sheet, 30% unordered region, 25% α-helix, 14% β-turn and 9% unknown structure (Fig. 4c). When EDTA was washed out and excess Ca^2+^ was added, the protein conformation was nearly restored to that of the native state, with 25% β-sheet, 28% unordered region, 21% α-helix, 13% β-turn and 13% unknown structure (Fig. 4d). These results indicate that Ca^2+^ increases the β-sheet and unknown structure composition. Previous reports have suggested that the unknown structure with a wavenumber of approximately 1610 cm^−1^ is associated with amyloid fibrils arranged by β-sheets^31^. Similarly, caddisfly silk, adhesive fibers produced by an insect, contains Ca^2+^-stabilized β-sheets, and reduction of β-sheets by EDTA reduced the mechanical properties of the silk^15^. These data demonstrate that EDTA treatment changes the conformation of byssal proteins by disturbing their arrangement and possibly by expanding nanocavities.

### Mechanical test of distal regions of threads

**Figure 5:**
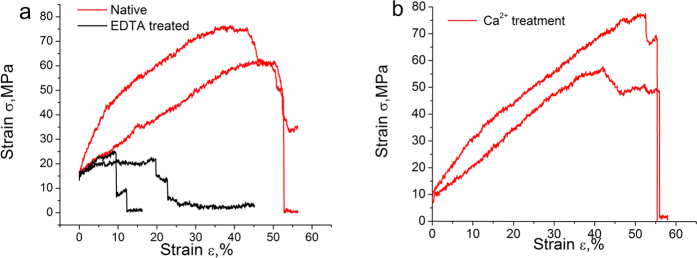
Mechanical performance of the byssus distal region. Stress-strain curves of (**a**) Native and EDTA-treated byssus of P. fucata, (**b**) EDTA-treated byssus after immersion in Ca2+ (Two typical curves of one sample are displayed. One-way ANOVA tests are performed, P < 0.05).

To determine whether the expanded cavities and the altered conformation of byssal proteins affected the mechanical properties of the byssus, stress-strain curves were acquired. The distal regions of native byssus showed a maximum stress of 67 MPa and a strain of 45% (Fig. 5a). Interestingly, the maximum stress (σ_M_) and the maximum strain (ε_M_) of the EDTA-treated byssus were significantly reduced to approximately 27 MPa and 23%, respectively (One-way ANOVA tests, P<0.05) (Fig. 5a). After immersion in Ca^2+^ for 10 h, σ_M_ and ε_M_ were restored to 64 MPa and 40%, respectively, nearly identical to the original values (Fig. 5b). Similarly, after immersion in Ca^2+^ for 10 h, the toughness and elastic modulus of EDTA-treated byssus also restored closely to the original values (Table S1). To compare *P. fucata* with well-studied byssus of other species, the byssus from *M. californianus*^32^, *M. edulis*^14^,^24^ and *Tridacna maxima*^14^ were analyzed (Table 2). The distal regions of *P. fucata* showed comparable tensile strength with *Mytilus sp.* (normalizing for the diameters of byssus) with lower extensibility, whereas the mechanical performance of the *P. fucata* byssus was overall stronger than that of *T. maxima*. These results indicated that EDTA treatment reduced the tensile strength and extensibility of the byssus, likely due to the aforementioned altered ultrastructure in both the cuticle and core regions of the byssus. In particular, Ca^2+^ appears to be the main metal ion responsible because it can reversibly change the byssal protein conformation, can alter the size of nanocavities, and can change the mechanical properties. In *Mytulis* species, the byssus mechanical properties vary greatly from the proximal to the distal region^32^. We think it is possible that it is the same case in *P.fucata*. However, because the focus of this work is the distal region, we did not test the mechanical properties of proximal region in *P.fucata*, which should be studied in the future.

### Compositional analysis of distal regions of the byssus by XPS and ICP

**Figure 6:**
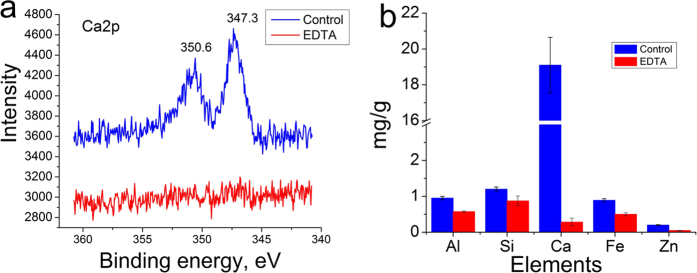
(**a**) X-ray photoelectron spectroscopy analysis of Ca in the distal region before and after 200 mM EDTA treatment. (**b**) Quantitative element analysis of distal regions using ICP.

Because metal ions such as Ca^2+^ are important for byssus function, the inorganic components of the distal regions were systematically examined. XPS investigates the surfaces of a sample, and cuticle analysis and high-resolution XPS data yield information regarding atom valence. ICP precisely quantifies inorganic elements from a sample. For XPS, aluminum (Al), silicon (Si), calcium (Ca), iron (Fe), zinc (Zn), and the non-metal carbon (C) were chosen for analysis. In the Ca spectrum, two peaks at 350.6 and 347.3 eV were assigned to Ca 2p 1/2 and Ca 2p3/2 orbits (Fig. 6a). The high-resolution scan of the Al spectrum revealed a peak at 74.3 eV, which could be assigned to Al (III) 2p (Figure S5). In the Si spectrum, a peak was observed at 102.5 eV that belonged to Si (IV) 2p (Figure S5). The peak at 712.5 eV was assigned to Fe (III) 2p3/2, and a weak peak at 1022.0 eV appeared in the Zn spectrum, which was attributed to Zn (II) 2p (Figure S5). In agreement with the XPS data, quantitative analysis by ICP showed that Al, Si, Ca, Fe and Zn decreased from approximately 0.95, 1.2, 19.0, 0.89 and 0.20 mg·g^-1^ to 0.58, 0.88, 0.21, 0.50 and 0.050 mg·g^−1^, respectively (Fig. 6c). These data demonstrate that Ca is the most abundant inorganic element in the byssus. Previously, various metals are commonly found in the byssus across different species. For example, Si and Al are also found in the mussel byssus^33^. However, the roles they play are currently unknown. A previous study has shown that EDTA depleted iron and calcium ions in the blue mussel byssus and resulted in a 50% reduction in byssus hardness^26^. Therefore, it is reasonable to hypothesize that Ca^2+^ interacts with byssal proteins and stabilizes β-sheet structures, which correlates with byssus tensile strength and extensibility. By elemental analysis, complex elements were found in the byssus that may be related to its diverse functionality. However, the exact interaction between inorganic parts and byssal proteins awaits further investigation.

### Compositional analysis of distal regions of threads by proteomic methods

**Figure 7:**
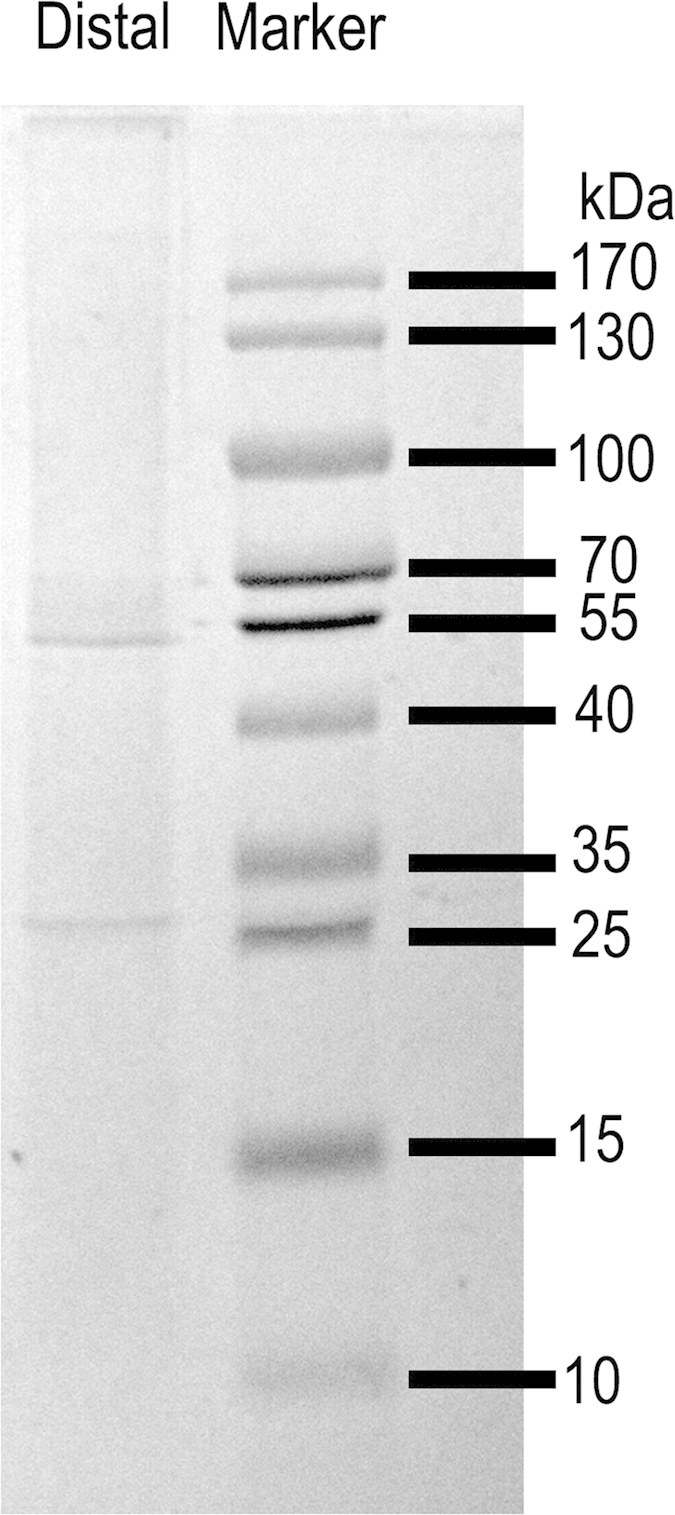
Insoluble byssal proteins separated on 12% SDS-PAGE and stained with Coomassie Brilliant Blue 250. Right pane: marker. Left pane: byssal proteins identified in the byssus extracts. Three prominent proteins bands and numerous faint proteins bands are observed.

While the inorganic components of the byssus have been examined, the organic components—the biomolecules constituting the byssus of *P. fucata*—remain uncharacterized. Effective high-throughput approaches to identify functional genes and proteins include RNA-seq and proteomic methods, respectively^16^. In the present study, proteins of the distal regions were first extracted and subjected to SDS-PAGE (Fig. 7). From the gel, three prominent protein bands were found at ~25, ~55 and >170 kDa. Besides, numerous faint protein bands were observed, which may be the degraded proteins. The faint protein bands are also found in the other extracted byssal proteins^34^. Since we run 1D SDS-PAGE, single protein bands may contain several components. Consequently, through LC-MS/MS analysis, four types of proteins (14 in total) were identified (Table 3): 1) proteins with repeated low-complexity domains; 2) enzymes, such as peroxidase, copper/zinc superoxide dismutase and proteinase inhibitors; 3) proteins associated with the cytoskeleton, such as actin, myosin and tubulin; and 4) other proteins, including foot protein 1 (P-FU1), P-FU2, calcium-transporting ATPase and ATPase. This diverse group of proteins demonstrates the intricate composition of threads, which are composed of structural proteins and some enzymes. In the mussel byssus, the main collagen proteins are precols. However, in the present study, we cannot identify collagen proteins by LC-MS/MS. The results are possibly caused by the fact that we use trypsin to digest protein extracts. Collagen in the extracts may have not enough cleavage sites like lysine or arginine, which makes them undetectable in the LC-MS/MS analysis. The limitation of this method is also encountered in identifying matrix proteins in shells^35^. To identify collagen, we may use other digest enzyme like pepsin or use RNA-seq method in the future. The presence of superoxide dismutase, peroxidase and proteinase inhibitors suggested a requirement by threads for preventing degradation by an oxidative environment or by proteinases from microorganisms in the seawater. This was a novel discovery, which should be considered in the future design of biomaterials. In addition, through the identification of these proteins, it was possible to obtain insights regarding the biological response of the bivalve byssus to its external environment. Surprisingly, the composition of proteins in the byssus of *P. fucata* displayed few similarities to structural proteins from other species based on sequence alignment. Even proteins extracted from byssus of zebra mussel^17^, green mussel^16^ and blue mussel differ to a large extent except few homologous proteins, suggesting different mechanisms of byssus extensibility across species.

### TSP-1 proteins

**Figure 8:**
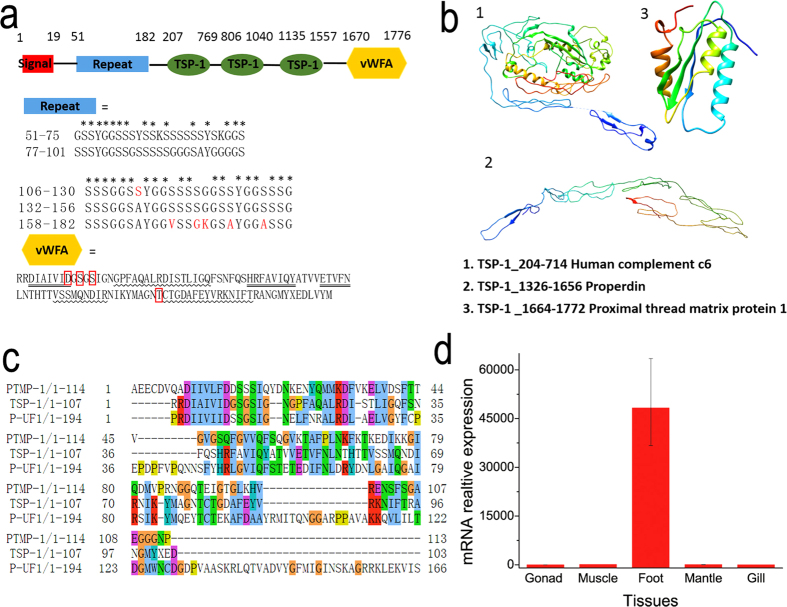
(**a**) Sequence analysis of TSP-1 (‘*’ represents the consensus amino acid residues; a red box indicates a conserved motif; ‘=’ is a β-sheet, and ‘~’ is an α-helix; TSP-1= Thrombospondin type 1 repeats, vWFA= von Willebrand factor A). (**b**) 3D structure of TSP-1 as predicted by Phyre2. (**c**) Sequence alignment of vWFA domains of the proximal thread matrix protein 1 (PTMP-1) of *Mytilus galloprovincialis*, as well as TSP-1 protein and P-UF1of *P. fucata* (sequence alignment was performed by the T-coffee method). (**d**) Tissue-specific gene expression of *TSP-1* by real time-PCR analysis.

Among all proteins identified in the distal region, a thrombospondin-1 protein (TSP-1) containing multiple domains was chosen for additional analysis by bioinformatics and real-time PCR. TSP-1 can be identified from the band 1 (>170kDa) and 2 (55-70kDa) in the PAGE gel (Fig. 7) through LC-MS/MS. Seven unique peptide fragments can be matched with TSP-1 (genome ID, pfu_aug1.0_532.1_65503.t1). Band 1 corresponds to TSP-1, which is in agreement with the predicted molecular weight of TSP-1 (191.6 kDa). Band 2 could be the degradation parts of TSP-1. TSP-1 contains a signal peptide, a low complexity repeat region, 24 TSP-1 repeats (three TSP-1 clusters in Fig. 8a), and a von Willebrand factor, type A (vWFA) domain (Fig. 8a). The low complexity repeat domain was highly enriched in GGSS, GSS and SS repeats, which were comparable to GGX repeats (X denotes a preference for alanine, leucine and phenylalanine) in the extensible flank regions of preCol-D^9^. Tyrosine contained in the repeats can be post-translationally modified to dopa, which can be useful for coordinating metals^6^. Nitro Blue Tetrazolium (NBT) assay shows the presence of dopa in the distal regions of byssus (Figure S7). Serine is also enriched in the repeats, which could be phosphorylated sites. Through phosphorylation prediction by NetPhos 2.0 (Figure S8), we found multiple possible phosphorylation sites especially at the serine residues in the repeats (residues 1–200, residues with scores above the threshold of 0.5 are likely phosphorylation sites). In caddisfly silks^15^, S is phosphorylated, which leads to strong electrostatic attraction between phosphorylated residues and Ca^2+^ ions. Therefore, it is possible that similar mechanism occurs here, which should be studied in the future. Upstream of the repeats and proximal to the C-terminus, 24 TSP-1 domains were found, which were hypothesized to be involved in a number of complement pathway proteins as well as extracellular matrix protein (IPR028499)^36^. Using Phyre2, 3D models of peptide from amino acid residues 204–714 and 1326–1656 were obtained (Fig. 8b1,2). These two parts showed similarity with human complement component c6 and the glycoprotein properdin with 100% confidence. Both proteins belong to a family of immune response proteins, suggesting a relationship between byssus formation and immune systems, which may present a fruitful avenue for future investigation. The vWFA domain is known to have a variety of functions, including intermolecular adhesion, cell migration, signaling, transcription and DNA repair. The binding of the domains with collagen occurred *via* the metal ion-dependent adhesion site (MIDAS) and involved three loops located on the upper surface of the molecule (IPR002035). MIDAS proved to be a potential candidate that might interact with the cations of byssal proteins. Interestingly, although the sequence of vWFA exhibited only 29% identity with the proximal thread matrix protein 1 (PTMP1) (E value = 7e-11)^29^,^37^, their 3D structures were predicted to be similar with 99.5% confidence (Fig. 8b3). From the sequence, we could identify the MIDAS motif (i.e., DXSXS…T, with X denoting any amino acid; Fig. 8a). The foot protein P-UF1 also contained this motif, as revealed by sequence alignment (Fig. 8c). However, the mechanisms underlying how Ca^2+^ interacts with byssal proteins and how Ca^2+^ is distributed in the byssus are currently unknown. Real-time PCR showed that the *TSP-1* gene was highly expressed (approximately 48000 times the level found in other tissues) in the foot, where the byssus was generated (Fig. 8d). Therefore, we inferred that TSP-1 is accountable for the byssus extensibility.

### Byssus extensibility in *P. fucata*: ultrastructure, Ca^2+^ and the TSP-1 protein

**Figure 9:**
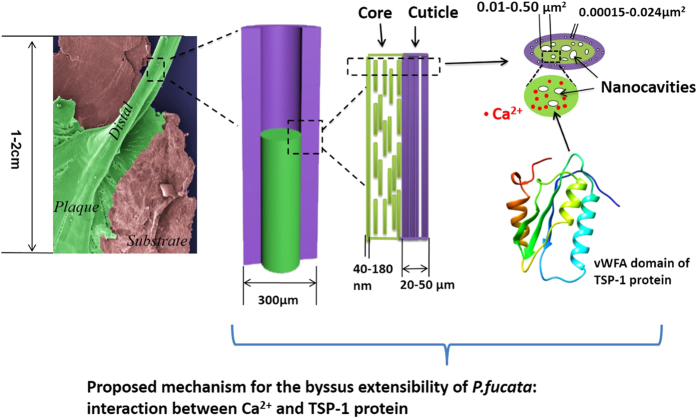
Byssus extensibility in *P. fucata*: ultrastructure, Ca^2+^ and the TSP-1 protein. The distal region of the byssus is made of cuticle and core. In the core, the protein fibrils are separated by nanocavities. In the cuticle, the protein fibrils are compact. Potential interaction between Ca^2+^ and the vWFA domain of TSP-1 protein increases the β-sheet conformation of byssal proteins and stabilizes the nanocavities between protein fibrils.

The distal region of the byssus (Fig. 9), which is the main extensible portion, is composed of a 20–50 μm cuticle and a 200–260 μm core. In the cuticle, protein fibrils are compact, whereas in the core, protein fibrils are separated by 0.01–0.50 μm^2^ nanocavities. Potential interactions between Ca^2+^ ions and the vWFA domain of TSP-1 protein may increase the β-sheet conformation of byssal proteins and stabilize the nanocavities between protein fibrils. Byssus extensibility is reduced when Ca^2+^ is sequestered by EDTA—an effect that can be reversed by the addition of Ca^2+^. Further studies attempt to characterize 1) the nature of the interaction between Ca^2+^ and the TSP-1 protein and 2) the function of other inorganic elements.

## Conclusions

In summary, we demonstrate the critical roles of Ca^2+^-stabilized nanocavities and TSP-1 proteins for byssus extension in *Pinctada fucata*. Byssus extensibility is hypothesized to be related to the interaction of byssal proteins with Ca^2+^ in the distal region of the byssus, which is composed of cuticle and a core containing nanocavities. The removal of Ca^2+^ by EDTA treatment partially reduces the β-sheet structure of byssal proteins and expands the nanocavities, resulting in the loss of extensibility. Proteomics and real-time PCR revealed the presence of thrombospondin-1 protein (TSP-1), which contains GGSS, GSS and SS repeats; thrombospondin-1 domains; and the metal ion interacting motif DXSXS…T. Moreover, the *TSP-1* gene is highly expressed in the foot of *P. fucata* and is inferred to be accountable for the extensibility of byssus. This study may shed light on the molecular mechanism underlying byssus extensibility and can guide the design of biomaterials in the future, particularly for use underwater.

## Additional Information

**How to cite this article**: Liu, C. *et al.* Extensible byssus of *Pinctada fucata*: Ca^2+^-stabilized nanocavities and a thrombospondin-1 protein. *Sci. Rep.* **5**, 15018; doi: 10.1038/srep15018 (2015).

## Supplementary Material

Supplementary Information

## Figures and Tables

**Table 1:** Nanocavity sizes before and after EDTA treatment (200 cavities were analyzed for size distribution).

Samples	Nanocavities with sizes over 0.025 μm^2^	
	Cuticle, Percent %	Core, Percent %
Native byssus	0	50
The whole byssus treated by EDTA	60	75
The thin section of byssus treated by EDTA	50	70

**Table 2:** Material properties of different byssus (σ_M_ is the average maximum tensile strength; ε_M_ is the average maximum extensibility until failure; d is the average diameter of byssus; five samples for each condition).

Samples	σ_M,_MPa	ε_M,_%	d, mm
Native byssus of *P. fucata*	66.9 ± 8.6	45.4 ± 5.8	~0.19
EDTA-treated byssus	27.2 ± 5.8	23.2 ± 16.0	~0.19
Ca^2+^ restored treated byssus	64.1 ± 12.0	40.2 ± 13.2	~0.19
Threads from *M. californianus*^32^	73.3 ± 5.7	109 ± 3	~0.25
Threads from *M. edulis*, fresh hydrated^24^	62.1 ± 8.9	>100	~0.13
Threads from *T*. *maxima*^14^	32 ± 9	28 ± 6	~0.10

**Table 3:** Proteins extracted from the byssus distal regions of *P. fucata* revealed by proteomic analysis.

Protein name	Protein ID in the genome	Protein homology	Domain
**TSP1**	pfu_aug1.0_532.1_65503.t1	Thrombospondin type 1 repeat containing protein [*Phytophthora cinnamomi*]	TSP1+vWFA
**P-UF1**	pfu_aug1.0_8925.1_31680.t1	Hypothetical protein LOTGIDRAFT_160181 [*Lottia gigantea*]	vWFA
**P-UF2**	pfu_aug1.0_7578.1_60273.t1	/	Coiled-coil
**Actin**	pfu_aug1.0_3120.1_51891.t1	Actin, cytoplasmic [*Crassostrea gigas*]	Actin
**Myosin**	pfu_aug1.0_13207.1_46613.t1	Myosin heavy chain, striated muscle [*Crassostrea gigas*]	Myosin head
**Tubulin**	pfu_aug1.0_158415.1_21306.t1	Beta tubulin, partial [*Yoldia scissurata*]	Beta_tubulin
**Peroxidase**	pfu_aug1.0_33687.1_48123.t1	Peroxidase-like protein; [*Pinctada margaritifera*]	An_peroxidase
**Peroxidase2**	pfu_aug1.0_385.1_65383.t1	Peroxidase-like protein; [*Pinctada margaritifera*]	An_peroxidase
**Copper/zinc superoxide dismutase**	pfu_aug1.0_2300.1_44326.t1	Superoxide dismutase [Cu-Zn] [*Crassostrea gigas*]	Sod_Cu
**Proteinase inhibitors**	pfu_aug1.0_69367.1_13096.t1	Hypothetical protein CGI_10023765 [*Crassostrea gigas*]	Thiol-ester_cl
**WR1**	pfu_aug1.0_1268.1_58418.t1	Hypothetical protein CGI_10019179 [*Crassostrea gigas*]	Worm-specific repeat type 1
**ATPase**	pfu_aug1.0_4962.1_67111.t1	Mitochondrial H+ ATPase a subunit [*Pinctada fucata*]	ATP-synt_ab
**ATP synthase subunit beta**	pfu_aug1.0_193784.1_28591.t1	Mitochondrial ATP synthase subunit beta [*Malleus albus*]	ATP-synt_ab
**Calcium-transporting ATPase**	pfu_aug1.0_1160.1_36844.t1	Sarco/endoplasmic reticulum calcium ATPase isoform A [*Pinctada fucata*]	E1-E2_ATPase

Notes: The protein ID is the gene ID in the draft genome of *Pinctada fucata,* which is available at http://marinegenomics.oist.jp/genomes/viewer?project_id=20&current_assembly_version=ver1.0.
