# Supplementary material for: Extensible byssus of Pinctada fucata: Ca2+-stabilized nanocavities and a thrombospondin-1 protein
Source: Sci Rep. 2015 Oct 8;5:15018. doi: 10.1038/srep15018 (PMC4597212; doi:10.1038/srep15018)
Supplement: Supplementary Information [file srep15018-s1.pdf]

Supporting Information

Extensible byssus of *Pinctada fucata*: Ca^{2+}-stabilized nanocavities and a thrombospondin-1 protein

Chuang Liu[1,2], *Shiguo Li*[1], *Jingliang Huang*[1], *Yangjia Liu*[1], *Ganchu Jia*[1],

Liping Xie[1*], *Rongqing Zhang*[1 *]

[1] Institute of Marine Biotechnology, Collaborative Innovation Center of Deep Sea Biology, School of Life Sciences, Tsinghua University, Beijing 100084 China,

E-mail: lpxie@mail.tsinghua.edu.cn (LX); rqzhanglab@mail.tsinghua.edu.cn (RZ)

[2] Tsinghua-Peking Joint Center for Life Sciences, School of Life Sciences, Tsinghua University, Beijing 100084 China

Table S1. Material properties of different byssus (five samples for each condition).

Samples	Toughness MJ • m^{-3}	Elastic modulus, MPa
**Native *P. fucata* byssus**	27.1±4.5	676±65
**EDTA-treated *P. fucata* byssus**	6.0±4.2	188±88
**Ca^{2+} restored *P. fucata* byssus**	25.0±4.7	285±62
**Mussel byssus (distal thread)[1]**	unknown	870
**Threads from *T. maxima* [1]**	unknown	495 ±85

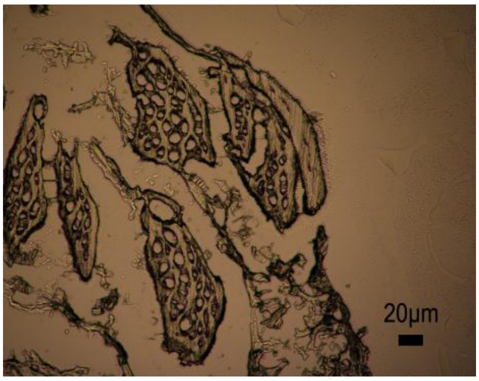

Figure S1. Optical photography of the byssus of *P.fucata*: the thin section (10μm) of transverse cross-section of proximal region

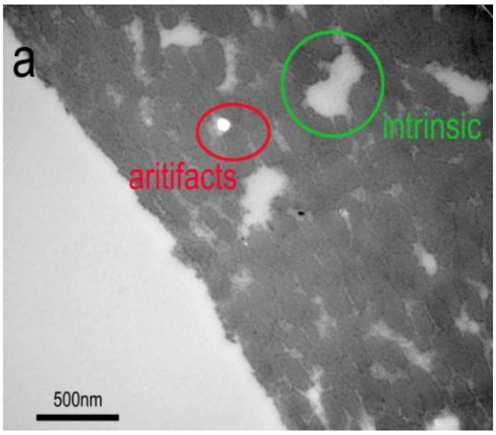

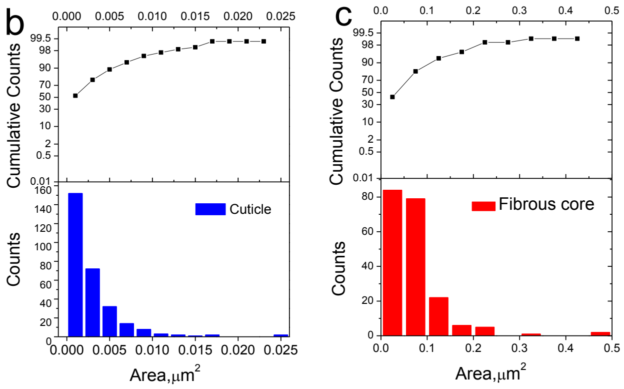

Figure S2. (a) TEM images of transverse cross-sections of distal region treated with 200mM EDTA in thin sections, in which the red circle represents the artifacts arising from incomplete penetration of resin while the green circle represents an intrinsic nanocavity in the sample. (b) and (c) are statistics of areas in the fibrous core and cuticle, respectively (counts are based on 200 cavities).

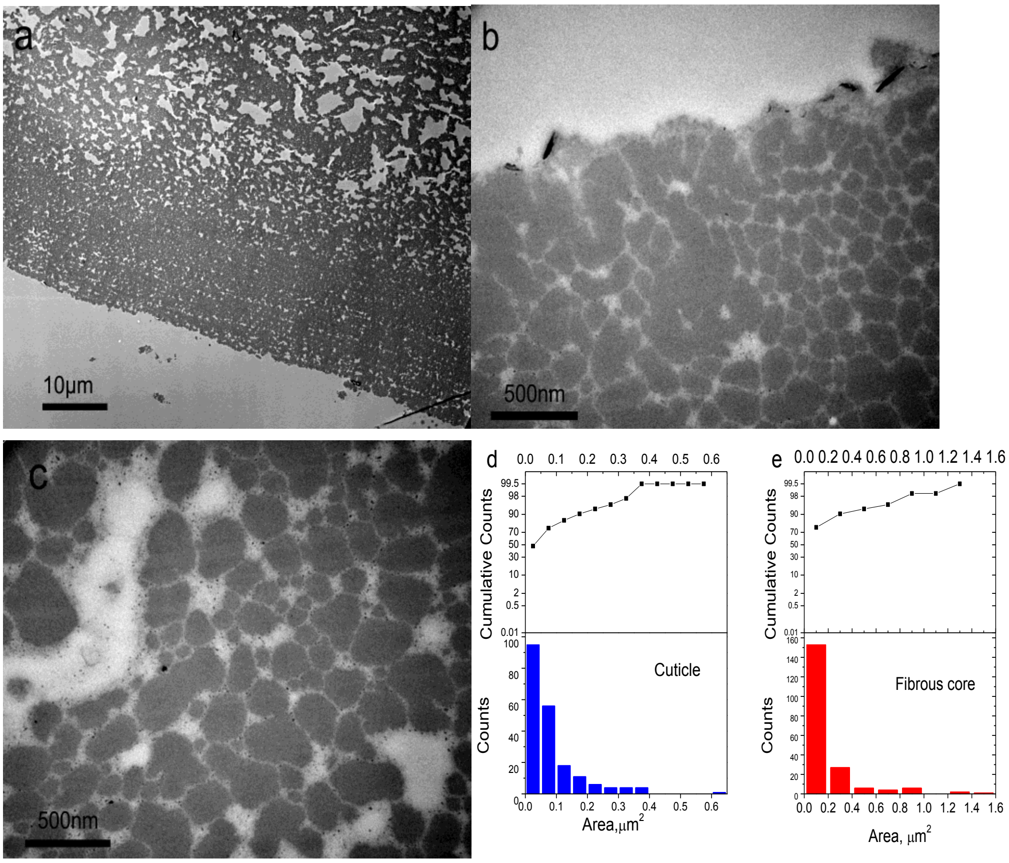

Figure S3. (a).TEM images of transverse cross-sections of distal region treated in EDTA for 12h; (b) and (c). High magnification of the cuticle and core regions shows expanded cavities. (d) and (e) are the corresponding areas distribution of cavities

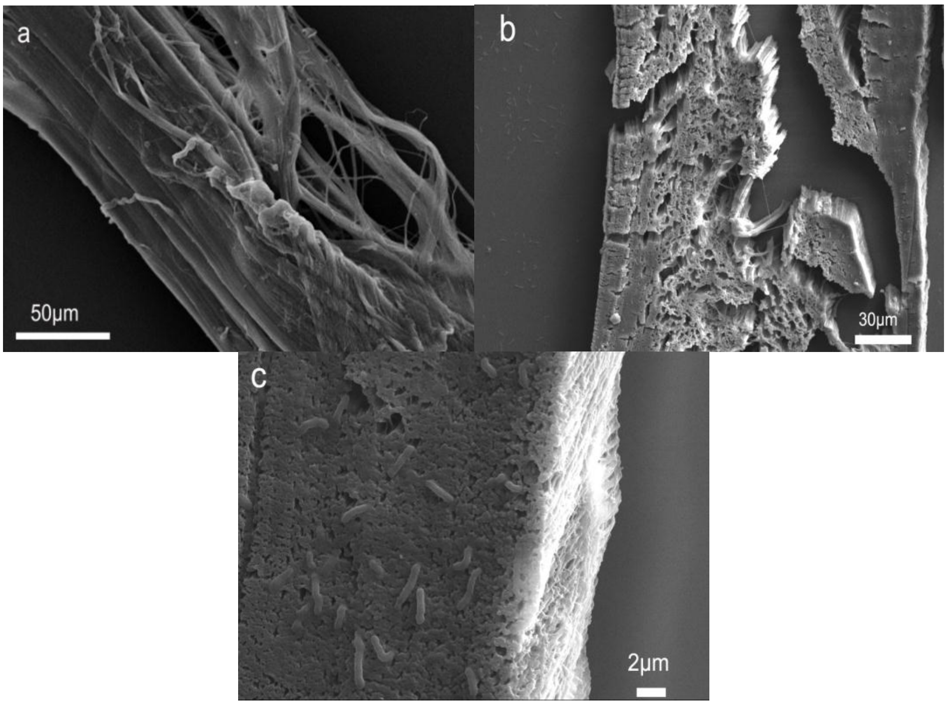

Figure S4. SEM of EDTA treated byssus: (a) transverse cross-section (b) cuticle region (c)

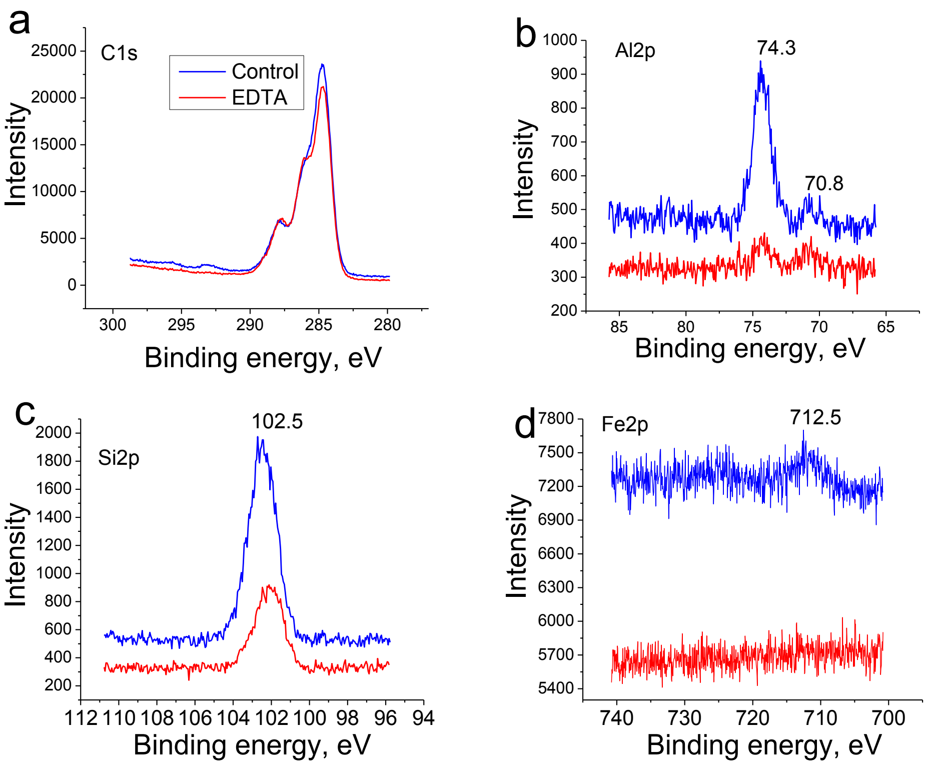

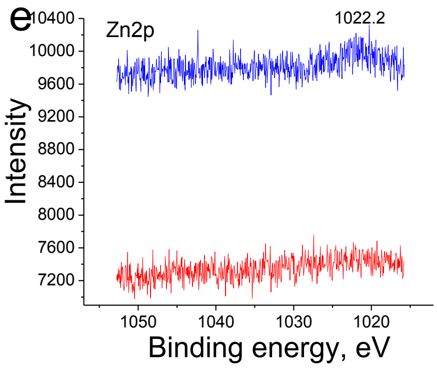

Figure S5. High resolution XPS analysis of C, Al, Si, Fe and Zn. Control, the pristine threads; EDTA, whole threads treated with EDTA for 12 hours

>pfu_aug1.0_532.1_65503.t1 TSP-1 protein

MKFTLALCLLVAGATVQGSYLSGFSSYYGKGGGSGGGGGSSYGSRSSYSSGS
SYGGSSSYSSKSSSSSSYSKGGSSSSSYGGSSGSSSSSGGGSAYGGGGSSSGGSS
SGGSSYGGSSSSGGSSYGGSSSGSSSSGGSAYGGSSSSGGSSYGGSSSGGSSSG
GSAYGGVSSGKGSAYGGASSGSAYGAASSGGSAYGKSSGSAYDCQVGPWSR
WSSPDAAGYSFRKRIVLLKAVNGGRPCPQPLVQKKIVPINCKVSAWSSWSAA
DDTGISKRSRRILIHPLNGGASCPNLGENRRVRIDCKVGPWSSWSGSGGVKSR
SRSVITYPQNGGSPCPDLHQVQKVPVDCCVGPWGDWGQPDSSGAVIRTREVT
TYPLNGGAECPDTVQRRKVKIDCVVGAWSSWSSPDSNGYSQRSRNIVTSPLN
GGRICPATHENRKVPIDCIVTNWLAWGQADSSGTQVRQRSIVRMSQNGGKG
CPDLAEKRKVKINCVVSAWGAWSAPNPSGMVIRSRHIIQHAMNGGNQCGPL
TETKPVPVNCVMSAWSAWTNMDASGVSRRTRVILVLPRNGGSECGKLSELR
KGPIDCVLSQWSAWSNSANTGSVSRTRFVVVAPQNGGKQCDKLLEIRKVKV
DCVVAPWCAWSKPDESGTSYRSRQIVRTALNGGKECPHLSEKRTVKINCVVS
QWSEYSAPNSAGIIIRTRSILQHPQNGGNQCPHLAESKKVAVDCLVCAWSKW
GETDSAGYQTRTRNVVRVNINGGKDCPALSERRKVPIDCVVSPWSKWSDPDP
ASGIVTRRRSILVHPQNGDPIDCKVSDWGEWGNPSNAGIRERRRGVIIHPFNG
GKECPPLVHRQKVPIDCVVSAWAAWGATAENGRTRRVRYVVIIPRNGGKEC
PNLSETKNVLLDCIVSPWGAWGPLNENGVSMRIRSVLRLPLNGGKLCPPLTE
KKNVPINCIVCTWSAWSTASDSGVQYRSRSIKTHPRNGGTNCPNLLERRKVAI
DCLVSEWGPFSAPDDSGVQTSIRTVLRVPRNGGKQCPELVRRRKVPINCKVS
DWAAWSDANEGGISVSTREILIHPLNGGDECPDLVRKRKVPIDCVVSEWSAFS
KPDAQGSSIRKRYVKRYNLNGGKECPHLIEAKKVACDCVVSDWAAWSVPNE
RGSSIRIRYIVKHPRNGGKQCPNLSERRRGPIDCKVSNWGQFSEPDEAGNIVRT
RSVLRFPQNNGKNCPDLVDKKKVPINCKVSSWSVWSDSNAAGQSSRFRTVLI
MPMNGGRECPDLVEKRIGAIDCVVSPWTEWGAVDGSGTIRRTRHIMTMPRN
NGKECPGLSEFRKVPRDCIVGAWGAWSQTDESGSQVRTRNVVTYPLNNGKQ
CPKLSQKRKIKIDCVVSPWGPWSATDNVGRSKRIRFVVRYPQNGGDRCPPLS
EGRQIPVDCRVSPWTEWTTADGQGISRRGRYVIQYQLNNGKKCPNLKEIRRV

NIDCRVSGWSPWTGADLNGDSFRFRSILIRPVNDGKSCPGLKERRKVPIDCVVSPWGSWSAPDPTGKSIRIRYVVRHPLNGGKDCPKLDQEKTVPIDCVLGPWSVWSESGEYGFVASERRVKRHALNGGKECERTSRRKRVTIDCVLGPWSEWGSPNNNGRARRSRQIIRKPIHGGKICGDIDESKIGPTPTPKQTAEHFAISFVKRLIDTRRDIAIVIDGSGSIGNGPFAQALRDISTLIGQFSNFQSHRFAVIQYATVVETVFNLNTHTTVSSMQNDIRNIKYMAGNTCTGDAFEYVRKNIFTRANGMYXEDLVYM

Peptide sequences	MH+, Da	Charge	Xcorr
YVIQYQLNNGKK	1468.77	2	3.66
YMAGNTCTGDAFEYVR	1854.77	2	3.59
SILIRPVNDGK	1211.71	2	3.45
NNGKECPGLSEFR	1508.68	2	2.99
EILIHPLNGGDECPDLVR	2047.02	2	2.54
HALNGGKECER	1271.57	2	2.42
YPQNGGDRCPPLSEGR	1803.80	3	2.01

Figure S6. Representative LC-MS/MS analysis: The protein derived from pfu_aug1.0_532.1_65503.t1 is TSP-1 protein through BLAST. Signal peptide predicted by SignalP 4.1 is underlined and the seven matched MS/MS peptides are marked pink; the table lists the corresponding parameters of peptides.

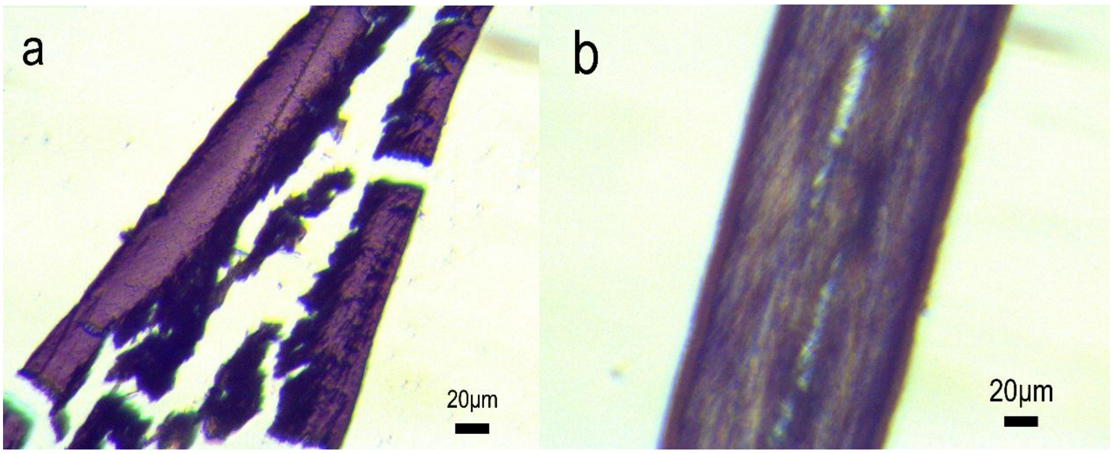

Figure S7. NBT/Glycinate staining indicates the existence of dopa in the byssal distal region. (a) and (b) are the cross-section and longitudinal section of distal region. The dopa in the threads of byssus was detected by staining with NBT/Glycinate (0.24 mmol/L NBT in 2mol/L potassium glycinate, pH 10) for 10min followed by rinsing twice with DI water.

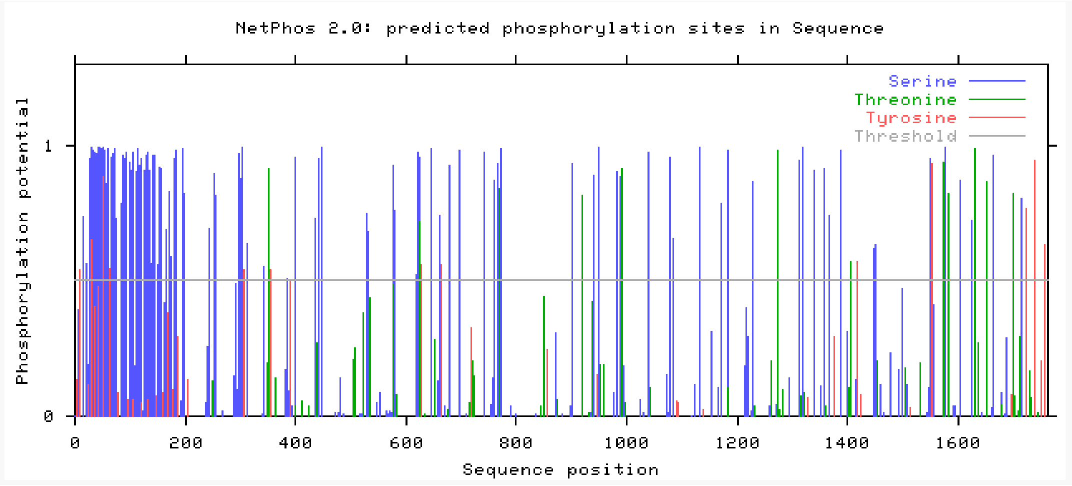

Figure S8. Prediction of phosphorylation sites of TSP-1 by NetPhos 2.0

Reference:

1. Miserez, A., Li, Y., Cagnon, J., Weaver, J.C. & Waite, J.H. Four-stranded coiled-coil elastic protein in the byssus of the giant clam, Tridacna maxima. *Biomacromolecules* **13**, 332-41 (2012).
